# Exploring the Potential of Transcranial Direct Current Stimulation for Relieving Central Post-Stroke Pain: A Randomized Controlled Pilot Study

**DOI:** 10.3390/life13051172

**Published:** 2023-05-12

**Authors:** Ji-Soo Baik, Jung-Hyun Yang, Sung-Hwa Ko, So-Jung Lee, Yong-Il Shin

**Affiliations:** 1 Research Institute for Convergence of Biomedical Science and Technology, Pusan National University Yangsan Hospital, Yangsan 50612, Republic of Korea; zisoo@pusan.ac.kr; 2 Department of Rehabilitation Medicine, Pusan National University Yangsan Hospital, Yangsan 50612, Republic of Korea; 3 Department of Rehabilitation Medicine, The Graduate School of Medicine, Pusan National University, Yangsan 50612, Republic of Korea

**Keywords:** transcranial direct current stimulation, central post-stroke pain, primary motor cortex, stroke

## Abstract

The potential of transcranial direct current stimulation (tDCS) as a non-invasive brain stimulation technique for treating pain has been studied. However, its effectiveness in patients with central post-stroke pain (CPSP) and the impact of lesion location remain unclear. This study investigated tDCS’s pain reduction effects in patients with CPSP. Twenty-two patients with CPSP were randomized into the tDCS or sham groups. The tDCS group received stimulation of the primary motor cortex (M1) for 20 min, five times weekly, for two weeks, and underwent evaluations at baseline, immediately after the intervention, and one week after the intervention. The tDCS group had no significant improvement compared to the sham group in pain, depression, and quality of life. Nevertheless, significant changes were identified within the tDCS group, and the pain trends appeared to be influenced by the lesion location. These findings provide important insights into the use of tDCS in patients with CPSP, which could inform further research and development of pain treatment options.

## 1. Introduction

Pain is an unpleasant physical sensation or emotional experience that can significantly affect the patient’s quality of life (QOL), motivation for treatment, and functional exercise [1]. Pain can be caused by tissue damage or inflammation; nonetheless, abnormal pain signals can also be delivered to the brain or spinal cord without direct damage or complications in the area [2,3]. Temporary pain can be managed with painkillers, or by treating the underlying damage or inflammation. On the other hand, neuropathic pain due to spinal cord injury, stroke, or multiple sclerosis is often chronic and challenging to completely cure with painkillers alone owing to the complex neuronal mechanisms [4,5,6]. Central post-stroke pain (CPSP) is a common condition [7] defined as pain that begins or is caused by a primary lesion or dysfunction of the central nervous system [8,9]. However, identifying CPSP’s specific pain mechanism is challenging due to its extensive patient variability [5]. Therefore, patients with chronic CPSP require long-term continuous treatment for pain management [10]. However, traditional treatments, such as anesthesia, surgery, and painkillers, have risks and side effects [11,12,13,14]. Therefore, alternative treatments that minimize side effects and maximize analgesic effects have been investigated [15,16,17].

Non-invasive brain stimulation (NIBS) techniques are other treatment methods that have been studied [17,18,19,20]. NIBS involves the magnetic or electrical stimulation of specific brain regions without surgical intervention. Repetitive transcranial magnetic stimulation (rTMS) and transcranial direct current stimulation (tDCS) are two NIBS techniques currently used in clinical settings [21,22,23]. These techniques stimulate the activity of neural network-related areas by releasing weak electrical currents or magnetic fields into the scalp. In addition, the stimulation selectively affects the activity of sodium and calcium ion channels in the local brain region [24,25,26].

Four main pain mechanisms are associated with CPSP [9,27]. The first is a dissociated sensory loss related to an imbalance in the integration of the lesioned spinothalamic tract and deafferentation. The second mechanism is central disinhibition, resulting from activating disinhibited thalamic reticular nuclei containing inhibitory interneurons at the thalamic level. The third mechanism involves overactive central pain-receptive neurons. The fourth mechanism involves an abnormal role of the cingulum [9]. The pain mechanisms associated with CPSP highlight the complex nature of this condition and the need for targeted treatments. NIBS techniques, such as tDCS and rTMS, are promising in reducing pain. Nonetheless, their effectiveness may vary depending on the pain mechanism and the lesion’s location in the brain. Previous studies reveal that tDCS and rTMS relieve pain after stroke [28,29,30]. In addition, rTMS may be more effective than tDCS in reducing pain; thus, using tDCS for pain modulation remains controversial [18,29,31] because previous studies have yielded conflicting results. However, tDCS has several advantages over rTMS, including portability and accessibility.

Therefore, this study aimed to clarify the potential benefits of tDCS in reducing pain in patients with CPSP compared to a sham group and explore the impact of lesion location on tDCS effectiveness.

## 2. Materials and Methods

### 2.1. Ethical Concerns and Study Design

This study was conducted until 31 December 2022. The institutional review board in Yangsan Pusan National University Hospital (IRB No. 03-2022-001) and the Korea Food & Drug Administration of the Korea Food & Drug Administration (approval number: b1268) approved this study. This randomized, single-blind, controlled pilot study was registered at ClinicTrials.gov (NCT0561216).

### 2.2. Participants and Procedures

The participants were enrolled through the following screening tests. The selection criteria for the participants were: (1) adults over 19 years of age; (2) those diagnosed with bleeding and first ischemic stroke through clinical observation and brain imaging; (3) those diagnosed with pain after stroke (CPSP); (4) individuals cognitive enough to understand and follow the researcher’s instructions; and (5) voluntary participation in the clinical trial and provision of written consent. Pain diagnosis after stroke was based on the following criteria: (1) a person with lesions in the cortex and subcortex due to bleeding and ischemic stroke regardless of the side (left, right brain, both sides, or one side); and (2) pain begins immediately after the stroke, and a person with a visual analog scale (VAS) pain score of ≥3 points in the body area corresponding to the lesion of the central nervous system [32].

Patients were excluded if one of the following criteria was met: (1) a fracture or orthopedic surgery in the pain or related region; (2) accompanying significant underlying diseases such as hypertension, arrhythmia, kidney disease, epilepsy, or cancer; (3) major psychiatric diseases such as major depressive disorders, schizophrenia, and dementia; (4) complex regional pain syndrome by the BUDAPEST Criteria [33]; (5) an etiology of other pain in the relevant area, such as skin disease or peripheral nerve damage; (6) pregnancy and lactation; (7) metals in the brain or body due to brain surgery; (8) anamnesis of convulsions; (9) use of similar devices within six months before this study; and (10) noncompliance to drugs that may affect pain, depression, and QOL during the study.

Participants were voluntarily recruited through research recruitment posters at the Yangsan Pusan National University Hospital. The doctor explained the research content to the participants to obtain consent. The doctor conducted interviews and screening tests and assessed the medical records to check for CPSP in patients with stroke and identify the selection criteria. Participants who met the inclusion criteria signed a consent form voluntarily and were enrolled in this study. Subsequently, a third party who did not participate in this study randomly assigned participants to the experimental (tDCS) and control (sham) groups at 1:1. The targeted number of participants was 22, which was calculated using G*Power Program 3.1 based on a previous study by Bae, et al., (effect size was 0.414, α was 0.05, and the (1-β) was 0.95) [34].

### 2.3. Intervention and Assessment

**Figure 1:**
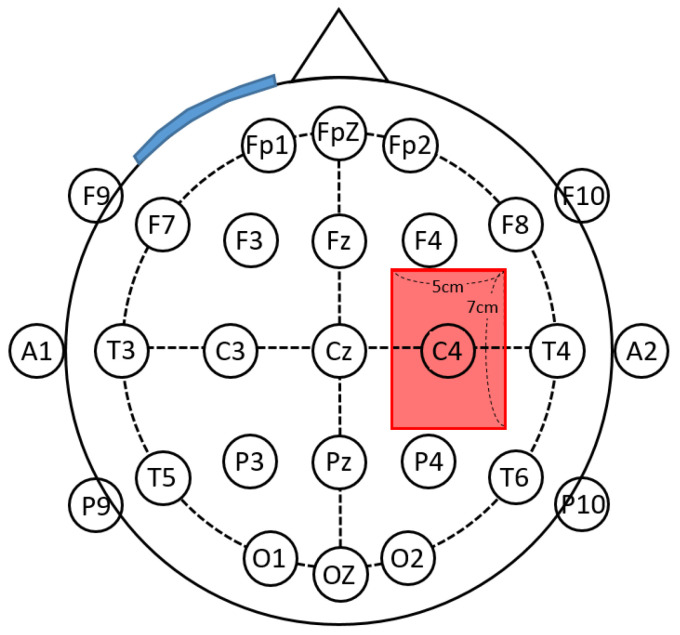
Electrode attachment location and size. Red electrode: M1; C3, C4 (primary motor cortex), Anode; blue electrode: over the contralateral orbitofrontal region, cathode.

**Figure 2:**
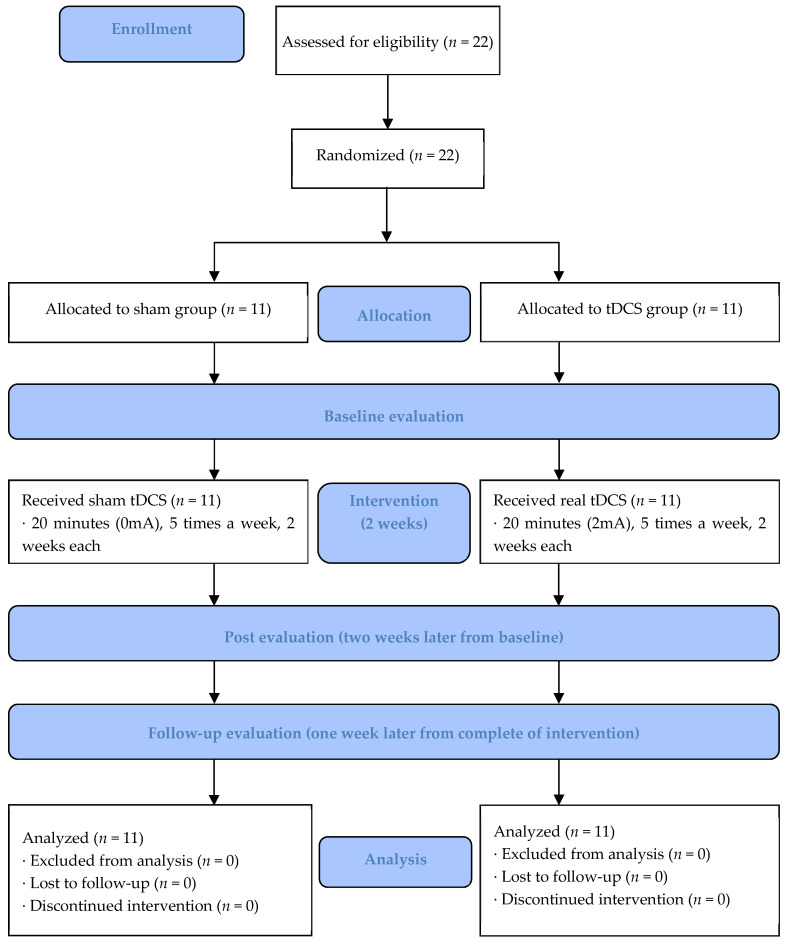
Flowchart of the study progress according to the CONSORT guidelines.

We used the DC-STIMULATOR PLUS [NeuroConn GmbH (Germany)/license number of the Korea Food & Drug Administration (A16180.02/3rd grade)] tDCS intervention method, based on the 10–20 System [35] targeting the primary motor cortex (M1; C3, C4- Anode, over the contralateral supraorbital region-cathode) (Figure 1) [36]. Per the method of other studies, the intervention lasted for 20 min (2 mA), five times weekly for two weeks [34]. The sham device was the same as the test device in the tDCS group; however, the electrical stimulus was blocked. Both groups underwent a post-intervention evaluation (T1) two weeks after the baseline (T0), and the follow-up evaluation (T2) was conducted one week after the intervention was completed (Figure 2).

The primary outcome was assessed using the brief pain inventory (BPI), which evaluated the strength of the overall pain [37]. The BPI evaluates how much pain has affected everyday life, mood, walking, relationships, sleep, and leisure activities and whether the pain for the last week or 24 h. The secondary outcome was assessed using Beck Depression Inventory (BDI) to evaluate depression [38]. The evaluation was based on a previous study that revealed the correlation between pain and depression [39]. The QOL was also reportedly associated with pain and depression; therefore, it was a secondary outcome [1,40]. Finally, the QOL was assessed using the Euro Quality of Life-5 Dimensions (EQ5D) [41].

The side effects were assessed each time tDCS was applied for safety evaluation. In addition, the degree of expression was recorded following the abnormal case evaluation criteria (mild, moderate, severe, and severe adverse event), and the causal relationship with the tDCS was evaluated.

### 2.4. Statistical Analysis

All statistical analyses were performed using the IBM SPSS Statistics version 23 (IBM Corp., Armonk, NY, USA). Repeated measures ANOVA was used to control baseline differences in the study group age and covariates and compare the assessment scores before and after the intervention. When analyzing group differences, covariance analysis was performed to rule out the influence of age, years of education, and cognitive assessment scores. Comparisons before the intervention were analyzed using the Wilcoxon signed-rank test. A *p*-value of < 0.05 was considered significant.

## 3. Results

### 3.1. General Characteristics of the Participants

#### 3.1.1. General Characteristics

All 22 people who were screened were registered in the study. The 22 participants in this study completed the intervention without dropping out. Eleven participants were in the tDCS group (six males and five females), and eleven were in the sham group (two males and nine females). No significant difference in age existed between the tDCS (56.00 ± 11.34 years) and the sham (59.91 ± 9.18 years) groups (*p* = 0.223, Z = −1.219). According to the research dates, no significant difference was observed in the onset period between the tDCS (27.64 ± 10.54 months) and the sham (29.18 ± 13.13 months) groups (*p* = 0.429, Z = −0.791). The most common mechanism of damage in patients with CPSP patients involved the basal ganglia (BG) (*n* = 11, 50%), followed by the cortex (*n* = 6, 27.3%), and the least common mechanism included the thalamus (*n* = 5, 22.7%). Table 1 provides an overview of the participants’ general characteristics.

#### 3.1.2. Clinical Symptoms and Pain Sites According to the CPSP Lesion

**Figure 3:**
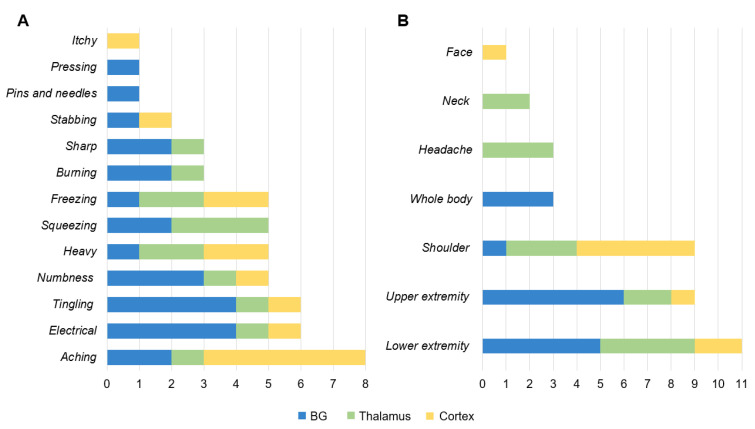
The figure indicates the clinical pain symptoms (**A**) and pain sites (**B**) according to the stroke lesion. Regarding clinical symptoms (**A**), all responses were based on duplicate choices. However, participants who selected “whole body” were not included in the “upper extremity” and “lower extremity” categories.

Figure 3 illustrates the clinical pain symptoms and sites according to the lesions in patients with CPSP. All clinical pain symptoms and sites were gathered through open-ended assessment questions and subsequently categorized into groups. The clinical symptoms reported based on the location of damage are illustrated in Figure 3A. The most commonly reported symptoms in the BG group were tingling (*n* = 4; 36.4%), electrical sensation (*n* = 4; 36.4%), and numbness (*n* = 3; 27.3%). In the thalamus group, a squeezing sensation (*n* = 3, 60%) was the most common, followed by freezing (*n* = 2, 40.0%), and heaviness (*n* = 2, 40.0%). On the other hand, aching (*n* = 5, 83.3%) was the most frequently reported condition in the cortex group.

The pain sites reported based on the damage location are indicated in Figure 3B. The most commonly reported pain sites in the BG group were the upper extremities (*n* = 6; 54.5%), including the arms or hands, and the lower extremities (*n* = 5; 45.5%), including the legs and feet. In the thalamus group, the most frequently reported pain site was the lower extremities (*n* = 4, 80.0%), the shoulder (*n* = 3, 60%), and the head (*n* = 3, 60%). In the cortex group, the shoulder was the most commonly reported pain site (*n* = 5, 83.3%).

### 3.2. Effects of Intervention

#### 3.2.1. Primary Outcome-Pain Profile

**Figure 4:**
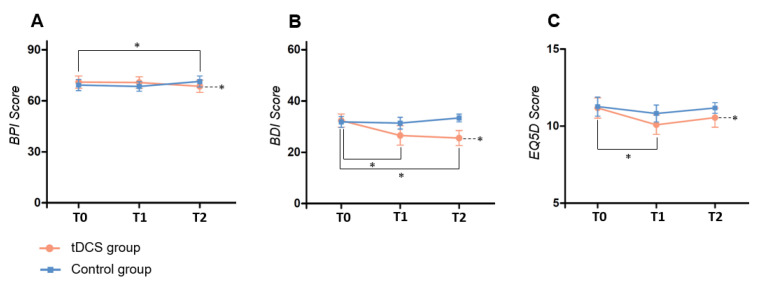
The graph above illustrates the evaluation score changes in the tDCS and sham groups at T0 (before intervention), T1 (immediately after intervention), and T2 (one week after completing the intervention) for BPI scores (**A**), BDI scores (**B**), and EQ5D scores (**C**). Notably, a downward trend in the EQ5D graph indicates an improved QOL, *p* < 0.05 *.

Figure 4A displays the BPI score change, representing the pain assessment for the tDCS and sham groups. No significant difference was observed in the BPI score change between the tDCS and sham groups from before the intervention (baseline; T0) to one week after completing the intervention (T2) (F_1, 20_ = 0.004, *p* = 0.950) (Appendix A). When the tDCS group was independently analyzed, the BPI scores according to repeated measures ANOVA between T0 and T2 changed significantly (F_1.35, 13.47_ = 4.264, *p* = 0.049). In the sham group, the repeated measures ANOVA did not reveal significant changes (Appendix A).

#### 3.2.2. Secondary Outcomes-Depression and QOL Profile

Figure 4B presents the BDI score changes for the tDCS and sham groups, a measure of depression. No significant difference existed in the BDI score change between the tDCS and sham groups between T0 and T2 (F_1, 20_ = 1.325, *p* = 0.263). However, when the tDCS group was independently analyzed, the BDI scores according to repeated measures ANOVA between T0 and T2 changed significantly (F_1.19, 11.93_ = 9.327, *p* = 0.008). In the sham group, the repeated measures ANOVA did not present significant changes (Appendix A).

Figure 4C illustrates the EQ5D score change in the QOL evaluation for the tDCS and sham groups. No significant difference was observed in the BDI score change between the tDCS and sham groups in the repeated measures ANOVA (F_2, 20_ = 0.379, *p* = 0.545) (Appendix A). When the tDCS group was independently analyzed, the EQ5D scores based on the repeated measures ANOVA between T0 and T2 changed significantly (F_2, 20_ = 8.195, *p* = 0.003). In the sham group, the repeated measures ANOVA did not reveal significant changes (Appendix A).

#### 3.2.3. The Tendency Analysis of tDCS’s Effect According to Stroke Lesion

The tDCS group (*n* = 11) was further divided into the BG (*n* = 5), thalamus (*n* = 3), and cortex (*n* = 3) groups to analyze the changes. Pain levels in the BG group decreased until T2. Pain levels in the thalamus group decreased at T1. However, a slight tendency for the pain to increase after completing the intervention existed. The pain levels in the cortex group increased at T1 but decreased at T2. However, none of the groups had substantial changes (Appendix B).

### 3.3. Side Effects

No special measures were required to address any side effects observed during the intervention. However, during the interview, four of the eleven participants in the tDCS group reported mild (level 0) side effects. Two of the four participants reported more severe spasticity and muscle spasms on the affected side during the intervention. Two participants reported sleeping more than usual, and one reported a headache. Despite these side effects, all participants completed the intervention and expressed no desire to discontinue the intervention. One week after completing the intervention, none of the participants reported any side effects during the follow-up assessments.

## 4. Discussion

We investigated the effectiveness of tDCS in reducing pain and depression and improving the QOL of patients with CPSP. We also explored potential differences in tDCS efficacy based on lesion location. Participants with CPSP were randomly assigned to the tDCS or sham group. The two groups had no significant differences regarding pain, depression, or QOL. However, positive trends were observed within the tDCS group. Additionally, there was no significant change within the sham group. Moreover, the exploratory analysis revealed that tDCS’s effects might vary depending on the lesion’s location, indicating the need for further investigation.

A previous study revealed that rTMS and tDCS have short-term effects on pain by modulating specific brain regions involved in pain perception [36]. However, their long-term effects on pain and effectiveness in treating CPSP are still being explored. Studies have yielded mixed results, with some reporting no significant improvement in pain reduction with tDCS compared to the sham group [42], which is consistent with the findings of this study. Further studies using various methodologies are required to fully understand the potential benefits of tDCS in CPSP. This study provides an additional exploration of the pain-modulating effects of tDCS in CPSP patients based on lesion location, which is a novel aspect not investigated in previous studies.

We studied the specific effects of tDCS stimulation on brain lesions located in the BG, thalamus, and cortex. As a result, we found no significant differences in pain, depression, or QOL between groups based on lesion locations following tDCS intervention. However, the pain score change graph revealed distinct patterns across different lesion locations, suggesting that tDCS may have various effects depending on the brain lesion’s location.

Patients with BG lesions experienced reduced pain immediately after the intervention and one week after completion. This is noteworthy because the BG is involved in motor control, facilitating the appropriate execution of movements and coordinating the activity of motor neurons [43]. When tDCS was applied to the M1 in Parkinson’s disease related to BG, a significant change was observed in upper limb function [44,45]. Therefore, it is expected that the tendency of pain reduction after TDCS in patients with BG damage in this study is related to the improvement of motor function. In fact, during the interview, two patients from the tDCS group (BG region) mentioned that their movements felt softer after the tDCS intervention.

Pain levels in patients with cortex lesions tend to increase immediately after tDCS but decrease one week after the intervention. This immediate increase in pain may be attributed to the temporary increase in muscle tone induced by tDCS stimulation, as two patients in the CA-tDCS group reported an increase in spasticity and muscle spasms during the tDCS intervention period. However, it is noteworthy that in an interview conducted one week after the completion of the intervention, these patients reported feeling a sense of lightness and smoothness in their movements. Previous research on the restoration of motor function in stroke patients has suggested that increased muscle tone can serve as an effective predictor of recovery from exercise-related damage [46]. In a recent study involving three patients, bilateral M1 tDCS showed inter-individual variability in improving upper limb pain and stiffness but demonstrated positive potential [47].

Patients with thalamic lesions experience slightly reduced pain immediately after tDCS. However, the pain levels increased again after one week. Previous studies have reported that the increase in cortical activity resulting from electrical stimuli can alleviate pain by spreading to the thalamic nucleus and inhibiting the bottom-up transmission of pain signals through the thalamus [48,49]. This study found that M1 tDCS did not have a sustained effect on patients with thalamic lesions after intervention completion. However, further analysis of the thalamus, cortex, and BG groups may have resulted from transient coincidence due to the limited number of participants. Therefore, caution should be exercised when generalizing the results of this study. Further research with a larger sample size is necessary to validate these findings.

Based on the discussion above, when investigating the effects of tDCS on pain in patients with CPSP, selecting the optimal stimulation site may depend on the specific brain lesion location. Thus, tailoring the stimulation to the patient’s lesion site may enhance the efficacy of tDCS in managing CPSP. This study’s results were not significant; however, this finding may be due to the limited sample size [50]. Nevertheless, tDCS resulted in different pain change patterns for each lesion. Therefore, further investigations are warranted to explore the potential of tailoring stimulation protocols based on individualized lesion characteristics. In addition, CPSP encompasses various disorders characterized by complex and multifactorial etiologies rather than a single underlying cause [31,51]. Therefore, a fine-grained treatment approach may be necessary to achieve maximum effectiveness in patients with chronic CPSP.

According to the secondary outcomes, there was no significant difference in depression and QOL between the two groups. However, a positive trend was observed within the tDCS group, whereas no significant change was observed within the sham group. Several studies have shown that tDCS can be an effective treatment for depression, and it has been found that there is a relationship between pain, depression, and QOL [52,53,54]. The dorsolateral prefrontal cortex (DLPFC), specifically the F3 position, is the most commonly studied tDCS location for depression [55,56]. However, in this study, M1 tDCS was used, and the experimental tDCS group experienced significant improvement in depression. This could be because M1 tDCS enhances physical exercise performance among patients with stroke, which may have implications for their mental health outcomes [50,57,58]. In addition, the antidepressant effects of M1 tDCS may be linked to its ability to enhance motor function. Consistent with this study’s findings, previous studies concerning the effects of DLPFC tDCS on depression and QOL also reported significant differences within the tDCS group. However, they did not find significant differences between the tDCS and sham groups [59]. Despite these diverse findings, further studies are necessary to clarify the potential therapeutic benefits of tDCS for depression and QOL outcomes.

Regarding safety and tolerability, this study reported that four individuals who underwent tDCS experienced increased somnolence or sleepiness. However, they could tolerate this side effect and expressed a desire to complete the intervention course. The findings of this study are consistent with those of previous studies that investigated the safety and tolerability of tDCS, as they documented similar side effects [60,61]. Notably, the observed symptoms were generally mild and did not interfere with the participant’s ability to complete the intervention. Safety and tolerability are critical considerations in administering any medical intervention, particularly for vulnerable patient populations or those with preexisting health conditions.

This study had a larger sample size than previous investigations; nonetheless, the number of participants still limits the generalizability and statistical power of the results [36]. Another limitation of this study is that while efforts were made to control for potential confounding variables, such as concomitant medication use and other treatments, external factors, such as environmental influences and individual neurophysiological changes, could have influenced the study results. Another potential limitation of this study is that the evaluation methodology did not incorporate objective measures of pain, such as physical or imaging assessments.

Despite the limitations of this study, the findings provide meaningful insights into the potential benefits and challenges associated with using tDCS to manage pain, depression, and QOL in patients with CPSP. Moreover, this study’s exploration of the differential effects of tDCS across different lesion locations may contribute to our understanding of individualizing treatment for this patient population. To obtain more robust and generalizable results on tDCS’s effects in patients with CPSP, future studies may benefit from utilizing a randomized controlled trial design with a larger sample size, longer intervention periods, and more detailed criteria for stratifying patient groups based on lesion location and other relevant factors. Additionally, incorporating objective measures of pain and other clinical outcomes may help reduce potential biases and increase the validity of the results.

## 5. Conclusions

In this study, we examined pain, depression, and QOL after tDCS was applied to the M1 area in patients with CPSP. We observed a significant difference within the tDCS group; however, the improvements were not significant compared to the sham group. Furthermore, our exploratory analysis of tDCS’s effects according to stroke lesions revealed that tDCS’s impact on pain in patients with CPSP differed according to the lesion’s location. This study’s results describe some possibilities regarding the clinical efficacy of tDCS for relieving pain and can predict patient reactions to tDCS stimulation. In addition, we believe that the discussions proposed in this study provide an overall framework for gaining insight into the differences in tDCS effects according to the lesions in patients with CPSP. Overall, this study contributes to developing effective and targeted treatment options for patients with CPSP.

## Figures and Tables

**Table 1:** General and medical characteristics of the participants.

Characteristics		tDCS (*n* = 11)	Sham-tDCS (*n* = 11)
Sex (*n* (%))	Male (*n* = 8)	6 (54.5)	2 (18.2)
	Female (*n* = 14)	5 (45.5)	9 (81.8)
Age (year) (median [IQR])		55.00 (48.00–62.00)	60.00 (55.00–61.00)
Onset (month) (median [IQR])		30.00 (24.00–32.00)	32.00 (19.00–39.00)
Affected side (*n* (%))	Right	5 (45.5)	6 (54.5)
	Left	6 (54.5)	4 (36.4)
	Multifocal	0 (0)	1 (9.1)
Pathological sites (*n* (%))	Basal ganglia (*n* = 11, 50.0%)	5 (45.5)	6 (54.5)
	Thalamus (*n* = 5, 22.7%)	3 (27.3)	2 (18.2)
	Cortex (MCA or MCA + ACA or MCA + PCA) (*n* = 6, 27.3%)	3 (27.3)	3 (27.3)
Pain symptoms (*n* (%))	Aching (*n* = 8, 15.7%)	3 (11.1)	5 (20.8)
	Electrical (*n* = 6, 11.8%)	3 (11.1)	3 (12.5)
	Tingling (*n* = 6, 11.8%)	3 (11.1)	3 (12.5)
	Numbness (*n* = 5, 9.8%)	3 (11.1)	2 (8.3)
	Heavy (*n* = 5, 9.8%)	4 (14.8)	1 (4.2)
	Squeezing (*n* = 5, 9.8%)	4 (14.8)	1 (4.2)
	Freezing (*n* = 5, 9.8%)	3 (11.1)	2 (8.3)
	Burning (*n* = 3, 5.9%)	1 (3.7)	2 (8.3)
	Sharp (*n* = 3, 5.9%)	1 (3.7)	2 (8.3)
	Stabbing (*n* = 2, 3.9%)	1 (3.7)	1 (4.2)
	Pins and needles (*n* = 1, 2.0%)	1 (3.7)	0 (0)
	Pressing (*n* = 1, 2.0%)	0 (0)	1 (4.2)
	Itchy (*n* = 1, 2.0%)	0 (0)	1 (4.2)
Pain site (*n* (%))	Lower extremity (*n* = 11, 28.9%)	6 (28.6)	5 (29.4)
	Upper extremity (*n* = 9, 23.7%)	5 (23.8)	4 (23.5)
	Shoulder (*n* = 9, 23.7%)	5 (23.8)	4 (23.5)
	Whole body (*n* = 3, 7.9%)	1 (4.8)	2 (11.8)
	Headache (*n* = 3, 7.9%)	2 (9.5)	1 (5.9)
	Neck (*n* = 2, 5.3%)	1 (4.8)	1 (5.9)
	Face (*n* = 1, 2.6%)	1 (4.8)	0 (0)

Abbreviations: IQR (Interquartile range), MCA (Middle cerebral artery), ACA (Anterior cerebral artery), PCA (Posterior cerebral artery). Duplicate responses were allowed for pain symptoms and sites.

## Data Availability

Due to the nature of this research, participants of this study did not agree for their data to be shared publicly, so supporting data is not available.

## Appendix A. Scores of BPI, BDI and EQ5D in the tDCS and Sham Groups

		**Mean(SD)**			**ANOVA with Repeated** **Measures T0, T1, and T2**		**ANOVA between Groups**	
		**T0**	**T1**	**T2**	**F**	***p***	**F**	***p***
BPI	tDCS	71(11.97)	70.36(11.36)	68.55(11.97)	4.264	0.049 *	0.004	0.950
	Sham	69.27(10.88)	68.45(9.16)	71.36(10.88)	0.411	0.548		
BDI	tDCS	32.36(8.54)	26.55(12.43)	25.55(9.72)	9.327	0.008 *	1.325	0.263
	Sham	31.82(7.25)	31.36(7.61)	33.36(4.97)	0.583	0.567		
EQ5D	tDCS	11.18(2.18)	10.09(2.07)	10.55(2.07)	8.195	0.003 *	0.379	0.545
	Sham	11.27(2.05)	10.82(1.83)	11.18(1.17)	1.265	0.304		
Abbreviations: T0 (before intervention), T1 (immediately after intervention), T2 (one week after completing the intervention), *p* < 0.05 *.								

## Appendix B. Scores of BPI, BDI and EQ5D for Each Stroke Lesion Group in the tDCS Group

		**Mean(SD)**			**ANOVA with Repeated** **Measures T0, T1, and T2**		**ANOVA between Groups**	
		**T0**	**T1**	**T2**	**F**	***p***	**F**	***p***
BPI	BG (*n* = 5)	75.20(15.04)	74.60(13.93)	72.40(13.61)	1.99	0.20	0.666	0.540
	Thalamus (*n* = 3)	70.33(11.50)	68.00(12.00)	69.67(14.01)	2.36	0.21		
	Cortex (*n* = 3)	64.67(5.13)	65.67(4.51)	61.00(5.00)	40.75	0.00 *		
BDI	BG (*n* = 5)	34.2(6.14)	25.8(9.58)	25(7.68)	6.68	0.02 *	0.021	0.979
	Thalamus (*n* = 3)	30(10.82)	29(15.72)	27.67(12.66)	4.54	0.09		
	Cortex (*n* = 3)	31.67(12.42)	25.33(18.15)	24.33(13.58)	0.29	0.77		
EQ5D	BG (*n* = 5)	11(2.55)	10(2.24)	10.4(2.07)	2.92	0.11	0.168	0.849
	Thalamus (*n* = 3)	12(2.00)	10.33(1.53)	11.33(1.53)	19.00	0.05		
	Cortex (*n* = 3)	10.67(2.31)	10(3)	10(3)	0.57	0.61		
Abbreviations: T0 (before intervention), T1 (immediately after intervention), T2 (one week after completing the intervention), *p* < 0.05 *.								
